# Bilateral Acute Angle-closure after Intraocular Surgery

**DOI:** 10.5005/jp-journals-10008-1173

**Published:** 2015-01-15

**Authors:** Kirsten Hoskens, Luis Abegão Pinto, Evelien Vandewalle, Nancy Verdonk, Ingeborg Stalmans

**Affiliations:** Resident, Department of Ophthalmology, UZ Leuven, Belgium; Auxiliary Professor, Department of Pharmacology and Neurosciences, Faculty of Medicine, Lisbon University, Lisbon, Portugal; Auxiliary Professor, Department of Ophthalmology, UZ Leuven, Belgium; Ophthalmologist, Department of Ophthalmology, Focus Eye Clinic, Belgium; Professor, Department of Ophthalmology, UZ Leuven, Belgium

**Keywords:** Acetazolamide, Choroidal effusion, Surgery complication, Acute secondary angle-closure.

## Abstract

We report the case of a 75-year-old woman who developed an acute bilateral angle-closure associated with choroidal effusion a day after an uneventful cataract surgery. The same patient had undergone a similarly uneventful cataract surgery two weeks before, under the same protocol, with no postoperative complication in the other eye. Medical treatment, including the use of oral sulfamide-related drugs (acetazolamide), topical beta-blockers and steroids led to a gradual decrease in intraocular pressure (IOP) and choroidal effusion. Despite initial reports suggesting a link between sulfamide-exposure and these rare forms of angle-closure, our report would suggest a more complex pathophysiology behind this intriguing phenomenon.

**How to cite this article:** Hoskens K, Pinto LA, Vandewalle E, Verdonk N, Stalmans I. Bilateral Acute Angle-closure after Intraocular Surgery. J Curr Glaucoma Pract 2014;8(3):113-114.

## INTRODUCTION

Choroidal swelling is a rare cause of acute angle-closure. Literature on this condition suggests the sudden cilio-choroidal effusion to be associated with an exposure to sulfa-related drugs, but more variables may be involved in this phenomenon.

## MATERIALS AND METHODS

**Figs 1A to D:**
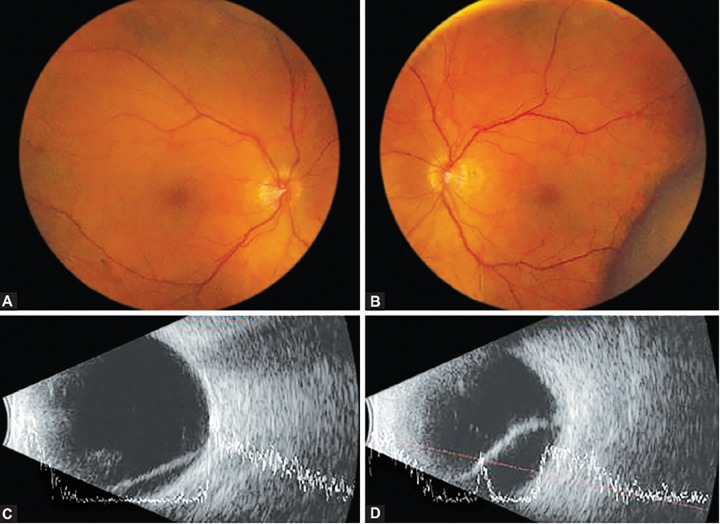
Fundus (A and B) and ultrasound (C and D) images at presentation, revealing a bilateral choroidal effusion

A 75-year-old nondiabetic, nonhypertensive woman presented with an acute bilateral increase in intraocular pressure (IOP)―35 mm Hg in the right eye (OD) and 37 mm Hg in left eye (OS)―after an uncomplicated left cataract surgery the day before. A bilateral decrease in visual acuity was present, due in part to a myopic-shift but also to a mild microcystic corneal edema. Gonioscopy showed a bilaterally closed angle without posterior synechiae, and fundoscopy revealed a bilateral bullous choroidal effusion (Fig. 1).

Two weeks back, the patient had undergone a similar procedure to the right eye, without postoperative complications. In both cases, the patient was premedicated with acetazolamide and postoperatively prescribed a combination of topical tobramycin, dexamethasone and diclofenac. The patient had neither known allergies nor prior adverse reaction to any topical or systemic medication.

## RESULTS

The patient was started on oral acetazolamide, topical beta-blocker and continued the topical steroid/antibiotic therapy. At day 2, the patient showed significant improvement in IOP (decreasing to 26 mm Hg and 22 mm Hg in OD and OS respectively). At day 5, IOP was 12 mm Hg OD and 13 mm Hg OS and uncorrected visual acuity improved to 0.0 logMar bilaterally. Choroidal effusion complete reabsorption was confirmed by sequential B-scans done during each visit. Gonioscopy revealed a bilateral open-angle, without synechiae.

## DISCUSSION

Most reports have suggested sulfamide-derivatives, such as acetazolamide or topiramate as the suspected culprit.^2,3^ However, our case highlights the caveats in the link between acetazolamide and these choroidal swelling events.^1,2,4^ First, a selection bias may exist, as acetazolamide is widely prescribed during the postoperative period. The retrospective nature of these reports further decreases the strength of this cause-effect relation. Furthermore, this drug has been safely used in the treatment of other drug-induced choroidal swelling.^5^

Interestingly, most cases relate to second ocular surgeries.^1,2^ Indeed, our patient had been administered acetazolamide peroperatively on both occasions, but only developed this reaction after the second surgery. It would seem that surgery-induced inflammation (which exists even in uncomplicated cataract surgery) could act as a sensitizer moment. A second-time contact with the same trigger could result in an immunological response.

## CONCLUSION

Our case suggests that the use of acetazolamide may be a safe and effective treatment of postoperative choroidal effusion and should not be ruled out as a treatment option.
